# Information Complexity of Time-Frequency Distributions of Signals in Detection and Classification Problems

**DOI:** 10.3390/e27100998

**Published:** 2025-09-24

**Authors:** Pavel Lysenko, Andrey Galyaev, Leonid Berlin, Vladimir Babikov

**Affiliations:** Institute of Control Sciences of RAS, 117997 Moscow, Russia; galaev@ipu.ru (A.G.); berlin.lm@phystech.edu (L.B.); babikov@ipu.ru (V.B.)

**Keywords:** information entropy, spectral complexity, time-frequency distribution

## Abstract

The paper considers the problem of detecting and classifying acoustic signals based on information (entropy) criteria. A number of new information features based on time-frequency distributions are proposed, which include the spectrogram and its upgraded version, the reassigned spectrogram. To confirm and verify the proposed characteristics, modeling on synthetic signals and numerical verification of the solution of the multiclass classification problem based on machine learning methods on real hydroacoustic recordings are carried out. The obtained high classification results (F1=0.95) allow us to assert the advantages of using the proposed characteristics.

## 1. Introduction

The history of signal detection and classification problems is associated with the development of processing methods, the main mathematical apparatus of which are signal transformations that transfer a time series from the time domain to another signal space. The most famous frequency conversion of signals is the Fourier transform, which has become de facto synonymous with frequency signal decomposition due to its mathematical validity and algorithmic efficiency. However, a strong limitation for the Fourier transform and any other frequency transformations remains their inability to track changes in the frequency components of the signal over time. To solve this problem, time-frequency transformations come to the rescue, the most popular of which is again the windowed Fourier transform and its square spectrogram, which is the simplest and most efficient time-frequency transformation. Cohen [1] introduced and described a general class of such transformations, special cases of which are the Wigner–Ville, Richachek, Choi–Williams transformations, etc. Thus, a method based on the Wigner–Ville transformation has become very popular for radar processing due to the convenience of using it to resolve linear frequency modulation (LFM) signals.

A comprehensive description of time-frequency transformations is contained in the monograph by Boilem Boashash [2]. In particular, this monograph, along with a number of the author’s other works [3,4,5] investigated the properties of the Rényi entropy for estimating the number of components of multicomponent signals, which also provides examples of using this estimate as a classification feature in signal classification problems. An original method for estimating the number of signal components based on entropy was also proposed by the current article’s authors in [6].

Since the basis of time-frequency distributions (TFDs) is the nuclear function generated by the reference signal, many authors compare TFDs with various nuclear functions on different signals, which is illustrated in the review [7].

In the case of an unknown reference signal, refs. [8,9] consider three variants of the distance between TFDs based on the Kullback–Leibler divergence, the Jensen–Shannon divergence, and the Rényi cross-entropy between the two distributions. Cross-entropy approach is also utilized in [10]. In turn, Baranyuk in [11,12,13] offers a modified formula for calculating the Jensen–Shannon divergence, taking into account the possible inconsistency of the TFD, where instead of the arithmetic mean, the geometric mean of two TFDs is used.

The problems of detecting mobile targets are considered in [14,15], where the Wigner–Ville entropy of the time-frequency distribution is calculated, and the decision to detect a target is made by exceeding the threshold of the information metric obtained based on the constant false alarm rate (CFAR) algorithm, which is an adaptive algorithm often used in radar to detect targets in conditions of noise, interference, and signal interference.

The work [16] explores the use of entropy in the problem of voice activity detection (VAD) for speech signal processing.

Entropic approaches to the processing of medical electroencephalogram (EEG) signals are investigated in [17], and in [18], the entropy from the Stockwell time-frequency transform (also known as the S-transform) is used to classify and detect heart valve pathology from electrocardiography (ECG) recordings.

In the articles [19,20], the entropy of TFD is used to solve problems of processing technical industrial signals related to monitoring the condition of production equipment.

The current work is devoted to the study of the concept of information complexity of time-frequency distributions in the problem of signal detection and classification. The novelty of the proposed approach is as follows:

- introduction of the concept of the TFD information complexity;
- using Rényi entropy to calculate the information complexity of two-dimensional probability distributions;
- application of the proposed information characteristics to the classification problem of acoustic signals.

The article consists of five main Sections, an Introduction, and a Conclusion. In Section 2 the classification problem of time series of acoustic origin is briefly formulated. In Section 3, Section 4 and Section 5, the necessary mathematical representations of time-frequency distributions are introduced, some of their previously unknown properties are investigated, and entropy and information criteria based on statistical complexity of various nature are proposed, as well as different ways for calculation of discrete distributions. Section 6 is devoted to a classification experiment of real natural and technical recordings using machine learning methods. In Conclusions (Section 7), the main results of the work are analyzed and plans for further research are outlined.

## 2. Statement of Signal Classification Problem

The problem of multiclass signal classification is based on a training dataset $X^{L}={\left\{x_{i},y_{i}\right\}}_{i=1}^{L}\subset X\times Y$ consisting of a set of objects ${\left\{x_{i}\right\}}_{i=1}^{L}\subset X$, where each object $x_{i}\in R^{2N}$ represents a studied signal of length 2N, and labels ${\left\{y_{i}\right\}}_{i=1}^{L}\subset Y$ such that $y_{i}\in \left\{1,2,\ldots ,K\right\}$, where *K* is the number of classes (K=2 corresponds to a binary classification, i.e., a detection problem when it is necessary to determine the presence or absence of an useful signal in a signal–noise mixture).

It is required to construct a mapping (parametric dependence model) a(x,β):X×A→Y with a vector β∈A of model parameters that would approximate the real unknown dependence f(x):X→Y with some criterion for minimizing empirical risk.

(1) $$\Gamma \left(\beta ,x_{i}\right)\to \min_{\beta },$$

where $\Gamma (\beta ,x_{i})$ is the loss function which indicates the deviation of a(x,β) from the correct answer.

The next step is the formation of a feature space. Each entry $x_{i}$ can be matched with basic signal characteristics: statistical features of a normalized/whitened signal, and a discrete Fourier spectrum

(2) $$DFT\left[k\right]=\sum_{n=1}^{2N}x_{i}\left[n\right]e^{-i2\pi nk/2N},$$

or the corresponding power spectrum

(3) $$F\left[k\right]={\left|DFT\left[k\right]\right|}^{2},$$

as well as various information characteristics based on two-dimensional time-frequency distributions presented in the following sections of the article. The purpose of the study is to examine the appropriateness of these characteristics for solution of the signal classification problem.

In various fields of physics and biology, the concepts of a useful signal, noise, and signal–noise mixture are defined independently. In hydroacoustics, which is the main application area of current study, environmental noise is random in nature and the useful signal is determined by the operation of ship mechanisms and is deterministic but unknown, and most importantly, the signal-to-noise ratio is available for indirect measurements.

## 3. Time-Frequency Distributions

### 3.1. Spectrogram and Wigner–Ville Distribution

Despite the limited time-frequency resolution, the simplicity of determining the spectrogram makes it one of the most popular time-frequency distributions, both for initial comparative theoretical analysis and as a reference in practical applications [2].

The short-term continuous Fourier transform of the signal x(t) is written as follows:

(4) $$STFT^{h}\left(t,\omega \right)=\int_{R}x\left(\tau \right)h\left(\tau -t\right)e^{-i\omega \tau }d\tau ,$$

where h(t) is some window function. The spectral power density, or the spectrogram *S*, corresponds to the square of the STFT value

(5) $$S\left(t,\omega \right)=|STFT^{h}{\left(t,\omega \right)|}^{2}.$$

The short-term discrete Fourier transform of a signal *x* with a window function *h* of length 2N is defined by the following expression:

(6) $$STFT^{h}\left[n,k\right]=\sum_{l=2Nn}^{2N(n+1)-1}x\left[l\right]h\left[l-2Nn\right]e^{-i2\pi kl/2N},$$

where 2N is the length of one window processed with DFT and *n* is the sequential number of the window. Consequently the discrete spectrogram is defined as

(7) $$S\left[n,k\right]=|STFT^{h}{\left[n,k\right]|}^{2},n=1,\ldots ,M,\;k=1,\ldots ,N,$$

where *M* is the total number of windows, and *N* is the total number of frequencies, taking into account the symmetry of the spectrum.

The Wigner–Ville distribution is a prototype of distributions that differ qualitatively from the spectrogram. Exploring its strengths and weaknesses has become one of the main directions in the development of this field. Wigner was aware of the existence of other joint densities, but chose the one that has now become the Wigner distribution “because it seems to be the simplest.” The Wigner distribution was introduced into signal analysis by Ville [1] about 15 years after Wigner’s paper. Ville argued for its plausibility and derived it using a method based on characteristic functions.

The Wigner–Ville distribution $W_{x}\left(t,\omega \right)$ for the signal x(t) is written as follows:

(8) $$W_{x}\left(t,\omega \right)=\int_{R}e^{-i\tau \omega }x^{*}\left(t-\frac{1}{2}\tau \right)x\left(t+\frac{1}{2}\tau \right)d\tau .$$

The Wigner distribution is considered bilinear in terms of the signal, since the signal is included twice in its calculation.

**Remark** **1.** *$W_{x}\left(t,\omega \right)$ will always have areas of negative values for any signal with one exception—for the LFM chirp signal with Gaussian amplitude. The reason is that for this signal, the Wigner distribution is not bilinear and belongs to the class of positive distributions.*

**Remark** **2.** *There is a connection between the spectrogram and the Wigner–Ville distribution through the window function. This relation connects two bilinear distributions, namely*

(9) $$S\left(t,\omega \right)=\frac{1}{2\pi }\int \int_{R^{2}}W_{x}\left(t^{\prime },\omega^{\prime }\right)W_{h}\left(t^{\prime }-t,\omega^{\prime }-\omega \right)\;dt^{\prime }\;d\omega^{\prime },$$

*where $W_{h}\left(t,\omega \right)$ is a Wigner–Ville transform of window function h(t).*

### 3.2. Reassigned Spectrogram

An important consequence of the (9) formula is that the value that the spectrogram takes at each point (t,ω) is the result of summing the values of $W_{x}$ within a certain time-frequency domain. In other words, S(t,ω) is a number that is assigned to the geometric center of the area being viewed over. For example, assigning the total mass of an object to its geometric center—an arbitrary point that, with rare exceptions, has no reason to correspond to the actual mass distribution.

A much more sensible choice is to assign the total mass to the center of gravity, and it is precisely this approach that corresponds to the Reassignment principle [2]:

- at each point (t,ω), where the value of the spectrogram is defined, two values are also calculated
  (10) $$\begin{array}{c} \hat{t}\left(t,\omega \right)=\frac{1}{S(t,\omega )}\int \int_{R^{2}}t^{\prime }W_{x}\left(t^{\prime },\omega^{\prime }\right)W_{h}\left(t^{\prime }-t,\omega^{\prime }-\omega \right)\;\frac{dt^{\prime }\;d\omega^{\prime }}{2\pi },\\ \hat{\omega }\left(t,\omega \right)=\frac{1}{S(t,\omega )}\int \int_{R^{2}}\omega^{\prime }W_{x}\left(t^{\prime },\omega^{\prime }\right)W_{h}\left(t^{\prime }-t,\omega^{\prime }-\omega \right)\;\frac{dt^{\prime }\;d\omega^{\prime }}{2\pi }, \end{array}$$
  which define the local distribution centers of $W_{x}$ through the window $W_{h}$ centered at (t,ω);
- then the value of the spectrogram is moved from the point (t,ω) to this centroid $\left(\hat{t}\left(t,\omega \right),\hat{\omega }\left(t,\omega \right)\right)$, which allows us to determine the reassigned spectrogram R(t,ω) as follows:
  (11) $$R\left(t,\omega \right)=\int \int_{R^{2}}S\left(t^{\prime },\omega^{\prime }\right)\delta \left(t-\hat{t}\left(t^{\prime },\omega^{\prime }\right)\right)\;\delta \left(\omega -\hat{\omega }\left(t^{\prime },\omega^{\prime }\right)\right)dt^{\prime }\;d\omega^{\prime },$$
  where δ is the Dirac delta function. In general, for $\left(t^{*},\omega^{*}\right)$, the value of the spectrogram $S(t^{*},\omega^{*})$ corresponds to a new position on the time-frequency plane, namely, moved to the point $\left(\hat{t}\left(t^{*},\omega^{*}\right),\hat{\omega }\left(t^{*},\omega^{*}\right)\right)$.

In practice, according to [21], to calculate the centroid $\left(\hat{t}\left(t,\omega \right),\hat{\omega }\left(t,\omega \right)\right)$, one can use a more efficient procedure based on STFT:

(12) t^(t,ω)=t+ReSTFTth(t,ω)STFT(t,ω),ω^(t,ω)=w−ImSTFTdh(t,ω)STFT(t,ω),

where $dh=\frac{dh}{dt}\left(t\right)$ is the derivative of the used window function h(t), and th=th(t).

## 4. Entropy of Time-Frequency Distributions

### 4.1. Classical Information Criteria and Discrete Distributions

In previous articles by the authors, the normalized Shannon entropy is studied, which is calculated from the square of the amplitude spectral distribution F[k] according to (3):

(13) $$H\left(P_{F}\right)=\frac{1}{\log_{2}N}\left(-\sum_{i=1}^{N}P_{F}\left[i\right]\log_{2}P_{F}\left[i\right]\right),$$

where $P_{F}\left[i\right]=\frac{F[i]}{\sum_{k=1}^{N}F\left[k\right]},\;i=1,\ldots ,N$ is a normalized discrete distribution so that $\sum_{i=1}^{N}P_{F}\left[i\right]=1$.

When calculating the sum (13), it is assumed that $\frac{0}{\log_{2}0}=0$ by continuity, and this assumption is valid for all subsequent equations.

This measure of entropy evaluates the uniformity of the distribution of signal energy in the frequency domain. High spectral entropy means greater uniformity in the distribution of signal energy, while low entropy means less uniformity. Spectral entropy can be used to discriminate a narrow band signal from a broadband one, for example, to distinguish between a tone signal and white noise; however, they cannot be used to distinguish two broadband signals, for example, a FM signal and noise.

In fact, time-frequency distributions concentrate energy for frequency-modulated signals in the same way that the Fourier transform concentrates energy for harmonic components. Thus, time-frequency extensions of entropy measures can distinguish between two different classes of broadband signals, while the energy of one class of signals can be evenly distributed in the time-frequency domain, for example, in the case of white noise, and the energy of the second class is concentrated in a certain area of the time-frequency plane, for example, in the case of a FM signal [2].

Time-frequency distributions, which are positive over the entire detection range, and normalized to the total energy, can be used to calculate information characteristics of a signal. In this case, the density of the discrete distribution will be determined by the formula

(14) $$P_{S}\left[n,k\right]=\frac{S[n,k]}{\sum_{n=1}^{M}\sum_{k=1}^{N}S\left[n,k\right]},$$

where the index *S* is associated with the spectrogram (7). In case of reassigned spectrogram (11), index *R* and notation $P_{R}$ for discrete distribution will be used later in the text.

The Shannon entropy of the time-frequency distribution is an extension of the spectral entropy, and can be obtained from the spectral entropy by replacing the Fourier transform of the signal with the time-frequency transform in Equation (13), and then by replacing the one-dimensional summation with a two-dimensional one. In the discrete case, it can be defined as

(15) $$H\left(P_{S}\right)=-\sum_{n=1}^{M}\sum_{k=1}^{N}P_{S}\left[n,k\right]\log_{2}P_{S}\left[n,k\right].$$

However, most time-frequency transformations do not have the property of positivity, so researchers prefer to use the Rényi entropy as an entropy measure

(16) $$H^{\left(\alpha \right)}\left(P_{S}\right)=\frac{1}{1-\alpha }\log_{2}\sum_{n=1}^{M}\sum_{k=1}^{N}P_{S}^{\alpha }\left[n,k\right],$$

where the index (α) defines the order of Rényi entropy (α>0,α≠1), usually α>1. When the parameter α tends to one, the Rényi entropy converges to the Shannon entropy.

According to [5], the value of the short-term Rényi entropy for a spectrogram of monocomponent harmonic signal is

(17) $$H^{\left(\alpha \right)}\left(P_{S}\right)=\log_{2}\left(\Delta t\right)+\left(\frac{1}{2\sqrt{\pi }\sigma_{w}}\right)\alpha^{-\frac{1}{2(1-\alpha )}}.$$

Equation (17) shows that the local entropy of the time slice of a single-component signal spectrogram depends on the duration of the interval Δt, the standard deviation $\sigma_{w}$ of the spectrogram window w(t), and of the order of α of the Rényi entropy. The local Rényi entropy decreases with increasing parameter $\sigma_{w}$. Large values of the entropy order emphasize the peak character of the spectrogram, which means they reduce entropy. Note also that the second term in Equation (17) decreases with increasing entropy order α, since $\lim_{\alpha \to \infty }\alpha^{-\frac{1}{2(1-\alpha )}}=1$. For a two-component signal, one can similarly obtain

(18) $$H^{\left(\alpha \right)}\left(P_{S}\right)\approx \log_{2}\left(\Delta t\right)+\left(\frac{1}{2\sqrt{\pi }\sigma_{w}}\right)\alpha^{-\frac{1}{2(1-\alpha )}}+1.$$

Hence follows the countable property of the Rényi entropy, i.e., the direct dependence of entropy on the number of harmonic components in the signal, which is a very powerful feature to determine its number.

### 4.2. New Information Criteria and Discrete Distributions

Another way to determine the discrete density is to normalize the columns of the matrix S[n,k] to the total energy of one signal processing window, as shown below:

(19) $$P_{SC}\left[n,k\right]=\frac{1}{M}\frac{S[n,k]}{\sum_{k=1}^{N}S\left[n,k\right]}.$$

Let us set the properties of the matrix $P_{SC}$.

**Remark** **3.** *$P_{SC}$ calculated by the formula (19) is a right stochastic matrix by construction if M=N.*

By the ergodic theorem, for a regular stochastic matrix, there exists a vector $\Pi =(\pi_{1},\ldots ,\pi_{M})$, $\sum_{n=1}^{M}\pi_{n}=1$ such that

$$\Omega \left(P_{SC}\right)=\lim_{j\to \infty }P_{SC}^{j}=1^{T}\left(\pi_{1},\ldots ,\pi_{M}\right),$$

where 1 is a unit vector.

**Remark** **4.** *The matrix $P_{SC}^{j}$ is a right stochastic matrix. Therefore, operations for calculating information characteristics, in particular the Shannon and Rényi entropy, are defined for this matrix.*

**Remark** **5.** *The Shannon and Rényi entropies can be calculated from the matrix* Ω *and the vector* Π*. It turns out that*

(20) $$\begin{array}{c} H\left(\Omega \right)=\frac{1}{2}\left(1+H(\Pi )\right),\\ H^{\left(\alpha \right)}\left(\Omega \right)=\log_{2}M+H^{\left(\alpha \right)}\left(\Pi \right). \end{array}$$

*At the same time, the maximum value of the Rényi entropy is $H^{\left(\alpha \right)}=2\log_{2}M$.*

It should be noted that for matrix elements, on average, ${P_{SC}}_{nk}\approx {P_{S}}_{nk}$ is valid for all n,k=1,…,M.

## 5. Complexity of Time-Frequency Distributions

In our earlier works [22,23], a characteristic called statistical complexity was investigated. The concepts of the disequilibrium function *D* and the statistical complexity *C*(*C* – complexity) of the discrete probability distribution *P* were first introduced in [24]:

C(P)=H(P)·D(P,Q),

where the disequilibrium D(P,Q) determines the distance between the signal distribution *P* and noise distribution *Q* and H(P) is the entropy of the signal distribution *P*.

The simplest example of a disequilibrium function is the Euclidean distance in the space of discrete probability distributions, which is convenient to use when evaluating and comparing with noise having a uniform spectral distribution $Q=\left\{\frac{1}{N},\ldots ,\frac{1}{N}\right\}$, is as follows:

(21) $$D_{SQ}\left(P\right)=\sum_{i=1}^{N}{\left(P\left[i\right]-\frac{1}{N}\right)}^{2}=\sum_{i=1}^{N}P{\left[i\right]}^{2}-\frac{1}{N}.$$

The statistical complexity, defined through the expression of the disequilibrium by (21), has the form

(22) $$C_{SQ}\left(P\right)=\frac{1}{\log_{2}N}\left(-\sum_{i=1}^{N}P\left[i\right]\log_{2}P\left[i\right]\right)\cdot \left(\sum_{i=1}^{N}{\left(P\left[i\right]-\frac{1}{N}\right)}^{2}\right).$$

Jensen–Shannon disequilibrium and corresponding statistical complexity are defined as

(23) $$\begin{array}{c} D_{JSD}\left(P\right)=JSD\left(P,Q\right),\\ C_{JSD}\left(P\right)=H\left(P\right)\cdot D_{JSD}\left(P\right), \end{array}$$

where JSD(P,Q) is Jensen–Shannon divergence

(24) $$JSD\left(P,Q\right)=H\left(m\right)-\frac{1}{2}(H\left(P\right)+H\left(Q\right)),\;m=\frac{P+Q}{2}.$$

The disequilibrium with total variation and corresponding statistical complexity are defined as

(25) $$\begin{array}{c} D_{TV}\left(P\right)=TV^{2}\left(P,Q\right)={\left(\sum_{i=1}^{N}|P\left[i\right]-\frac{1}{N}|\right)}^{2},\\ C_{TV}\left(P\right)=-\frac{1}{4\log_{2}N}\left(\sum_{i=1}^{N}P\left[i\right]\log_{2}P\left[i\right]\right){\left(\sum_{i=1}^{N}|P\left[i\right]-\frac{1}{N}|\right)}^{2}. \end{array}$$

Thus, transitioning from frequency distributions to time-frequency distributions naturally leads us to introduce and define a complexity function for them. Obvious candidates as a disequilibrium function for time-frequency distributions are as follows:

- Kullback–Leibler divergence $D_{KL}\left(P,Q\right)$;
- Rényi divergence $D^{\left(\alpha \right)}\left(P,Q\right)$ (can be considered a generalization of $D_{KL}\left(P,Q\right)$, since when α tends to 1, it becomes $D_{KL}\left(P,Q\right)$);
- Jensen–Shannon divergence for Rényi entropy $J^{\left(\alpha \right)}\left(P,Q\right)$;
- Euclidean distance $D_{SQ}\left(P,Q\right)$;
- Total signed measure of variation $D_{TV}\left(P,Q\right)$.

While the last two functions are trivial in terms of the transition to two-dimensional distributions, the first items of the list require some comments and are discussed in more detail below.

### 5.1. Rényi Divergence

The Rényi divergence between two time-frequency probability distributions *P* and *Q* has the form

(26) $$D^{\left(\alpha \right)}\left(P,Q\right)=\frac{1}{\alpha -1}\log_{2}\int \int P^{\alpha }\left(t,f\right)Q^{1-\alpha }\left(t,f\right)dtdf$$

and it becomes a Kullback–Leibler divergence when α tends to 1.

When $Q=\left\{\frac{1}{NM},\ldots \frac{1}{NM}\right\}$, one can write

(27) $$D^{\left(\alpha \right)}\left(P,Q\right)=\frac{1}{\alpha -1}\log_{2}\left(Q^{1-\alpha }\int \int P^{\alpha }\left(t,f\right)\right)dtdf=\log_{2}\left(NM\right)-H^{\left(\alpha \right)}\left(P\right).$$

A symmetrized divergence is often used. It is relatively straightforward to calculate and takes the following form:

(28) $$D^{\left(\alpha \right)}\left(P,Q\right)+D^{\left(\alpha \right)}\left(Q,P\right)=\frac{1}{1-\alpha }\left(\log_{2}\left(NM\right)+\alpha H^{\left(1-\alpha \right)}\left(P\right)-\left(1-\alpha \right)H^{\left(\alpha \right)}\left(P\right)\right).$$

In the current work, such a distance is not applicable as a disequilibrium, since in practice α>1 is used.

### 5.2. Jensen–Shannon Divergence for Time-Frequency Distributions

Richard Baraniuk et al. in their articles [11,12,13] introduced an analog of the Jensen–Shannon distance for TFDs

(29) $$J^{\left(\alpha \right)}\left(P,Q\right):=H^{\left(\alpha \right)}\left(\sqrt{PQ}\right)-\frac{H^{\left(\alpha \right)}\left(P\right)+H^{\left(\alpha \right)}\left(Q\right)}{2},$$

where $\sqrt{PQ}\left(t,\omega \right):=\sqrt{P(t,\omega )\cdot Q(t,\omega )}$, and the sign of “·” is an element-wise matrix multiplication. Now let us explore the properties of the function (29).

**Lemma** **1.** *It is valid for any discrete distributions P and Q for α>1*

(30) $$J^{\left(\alpha \right)}\left(P,Q\right)\geq 0,$$

*and equality is possible only if P=Q.*

**Proof.**  To prove the lemma, we need to evaluate the expression

$$\frac{{\left(\sum a_{i}b_{i}\right)}^{2}}{\sum a_{i}^{2}\sum b_{i}^{2}}\leq 1,$$

which is the square of the Cauchy–Bunyakovsky inequality, and $a_{i}$ and $b_{i}$ are the *i*-th elements of the corresponding distributions *P* and *Q* to the power of α/2. By applying the −log operation to this inequality, we obtain the statement of the lemma. □

**Lemma** **2.** *Let $P=\;∥p_{ij}∥$, i=1,…N, j=1,…M be valid for discrete densities P and Q, and Q=P+δρ, where $\delta \rho =\;∥\delta \rho_{ij}∥$, $\delta \rho_{ij}\ll 1$ and $\delta \rho_{ij}/p_{ij}\ll 1$. Then*

(31) $$J^{\left(\alpha \right)}\left(P,Q\right)=-\frac{1}{4}\frac{\partial H^{\left(\alpha \right)}}{\partial p_{ij}}\frac{{\left(\delta \rho_{ij}\right)}^{2}}{p_{ij}}+{o(∥\delta \rho ∥}^{2}).$$

**Proof.**  Let us decompose the function of many variables $H^{\left(\alpha \right)}\left(P\right)$ into a Taylor series up to the third term in the neighborhood of the matrix *P*. Here and further, a summation is performed using repeated indexes. We get

(32) $$H^{\left(\alpha \right)}\left(Q\right)=H^{\left(\alpha \right)}\left(P\right)+\frac{\partial H^{\left(\alpha \right)}}{\partial p_{ij}}\delta \rho_{ij}+\frac{1}{2}\frac{\partial^{2}H^{\left(\alpha \right)}}{\partial p_{ij}\partial p_{kl}}\delta \rho_{ij}\delta \rho_{kl}+{o(∥\delta \rho ∥}^{2}).$$

In turn, decomposing the function $H^{\left(\alpha \right)}\left(\sqrt{PQ}\right)$ into a Taylor series in the vicinity of the same point gives

(33) $$H^{\left(\alpha \right)}\left(\sqrt{PQ}\right)=H^{\left(\alpha \right)}\left(P\right)+\frac{1}{2}\frac{\partial H^{\left(\alpha \right)}}{\partial p_{ij}}\delta \rho_{ij}-\frac{1}{4p_{ij}}\frac{\partial H^{\left(\alpha \right)}}{\partial p_{ij}}{\left(\delta \rho_{ij}\right)}^{2}+\frac{1}{4}\frac{\partial^{2}H^{\left(\alpha \right)}}{\partial p_{ij}\partial p_{kl}}\delta \rho_{ij}\delta \rho_{kl}+{o(∥\delta \rho ∥}^{2}).$$

Subtracting the penultimate expression from the last one gives the statement of the lemma. □

Let us consider an elementary example to illustrate Lemma 2.

**Example** **1.** *Let us choose*

(34) $$\begin{array}{c} N=3,\\ M=1,\\ \alpha =2,\\ P=(1/3,1/3,1/3),\\ Q=(1/4,1/4,1/2),\\ \delta \rho =(-1/12,-1/12,1/6). \end{array}$$

*Then $\sqrt{PQ}=\left(1/\sqrt{12},1/\sqrt{12},1/\sqrt{6}\right)$. The calculation of entropies and divergence gives*

(35) $$\begin{array}{c} H^{\left(2\right)}\left(P\right)=\log_{2}3,\\ H^{\left(2\right)}\left(Q\right)=\log_{2}8-\log_{2}3,\\ H^{\left(2\right)}\left(\sqrt{PQ}\right)=\log_{2}3,\\ J^{\left(2\right)}\left(P,Q\right)=\log_{2}3-\frac{1}{2}\log_{2}8=\frac{\ln3}{\ln2}-\frac{3}{2}\approx 0.0849. \end{array}$$

*On the other hand, according to Lemma 2 we have $\nabla H^{\left(2\right)}=-\left(2,2,2\right)$ and*

$$J^{\left(2\right)}\left(P,Q\right)=\frac{2}{4\ln2}\left(\frac{3}{12^{2}}+\frac{3}{12^{2}}+\frac{3}{6^{2}}\right)=\frac{1}{18\ln2}\approx 0.0802.$$

*As one can see, the calculations of $J^{\left(2\right)}\left(P,Q\right)$ head-on using the formula (29) and Lemma 2 are close.*

### 5.3. New Information Characteristics

Summing up the discussion of the divergences of TFDs from previous Sections, it is proposed to investigate the following signal characteristics for signal classification:

1. Related to Shannon entropy:
  (36) $$\begin{array}{c} H\left(P\right)=\frac{1}{\log_{2}N}\left(-\sum_{i}P\left[i\right]\log_{2}P\left[i\right]\right),\\ C_{SQ}\left(P\right)=H\left(P\right)\cdot \left(\sum_{i}{\left(P[i]-Q[i]\right)}^{2}\right),\\ C_{JSD}\left(P\right)=H\left(P\right)\cdot JSD\left(P,Q\right),\\ C_{TV}\left(P\right)=\frac{1}{4}H\left(P\right)\cdot {\left(\sum_{i}\left|P[i]-Q[i]\right|\right)}^{2}. \end{array}$$
2. Related to Rényi entropy:
  (37) $$\begin{array}{c} H^{\left(\alpha \right)}\left(P\right)=\frac{1}{\log_{2}N}\left(\frac{1}{1-\alpha }\log_{2}\sum_{i}P^{\alpha }\left[i\right]\right),\\ C_{SQ}^{\left(\alpha \right)}\left(P\right)=H^{\left(\alpha \right)}\left(P\right)\cdot \left(\sum_{i}{\left(P[i]-Q[i]\right)}^{2}\right),\\ C_{JSD}^{\left(\alpha \right)}\left(P\right)=H^{\left(\alpha \right)}\left(P\right)\cdot JSD\left(P,Q\right),\\ C_{J^{\left(\alpha \right)}}^{\left(\alpha \right)}\left(P\right)=H^{\left(\alpha \right)}\left(P\right)\cdot J^{\left(\alpha \right)}\left(P,Q\right),\\ C_{TV}^{\left(\alpha \right)}\left(P\right)=\frac{1}{4}H^{\left(\alpha \right)}\left(P\right)\cdot {\left(\sum_{i}\left|P[i]-Q[i]\right|\right)}^{2}. \end{array}$$

Moreover, the discrete distributions *P* can be calculated using different supports and have different indexes accordingly:

1. $P_{F}$ for spectrum (3) one-dimensional discrete distribution;
2. $P_{S}$ for spectrogram (7) two-dimensional discrete distribution;
3. $P_{R}$ for reassigned spectrogram (11) two-dimensional discrete distribution.

**Remark** **6.** *Summation in (36) and (37) is performed:*

1. *by i=1,⋯,N for one-dimensional discrete distributions;*
2. *by i=[n,k] where [n,k] is a pair of indices for two-dimensional discrete distributions.*

Thus, systems (36), (37), and different discrete distributions present a significant number of information characteristics, which can be used as classification features and will be explored in the next section.

## 6. Modeling

This section is devoted to verifying of the theoretical proposals presented in the previous sections and the applicability of the proposed information characteristics to solve the problem of detection and classification in numerical experiments with both model signals and more complex real-world recordings.

### 6.1. Model Signal Description

To illustrate the obtained theoretical results, the following types of model signals are used:

- harmonic signals;
- linearly frequency-modulated chirp signals (LFM chirp signals);
- model signals of marine vessels.

The harmonic signal has the form

(38) $$x\left(t\right)=\sum_{n=0}^{K-1}A_{n}\sin\left(2\pi \left(f_{0}+n\Delta f\right)t\right),\;\;t\in \left[0,T\right]$$

and consists of the sum of *K* harmonic components with amplitudes $A_{n}$ and frequencies $f_{0}+n\Delta f$, where $f_{0}$ and Δf are the constant fundamental frequency and the step between the frequencies, respectively. The sampling frequency and window size are selected so that harmonic samples are not blurred in the resulting spectrum.

The LFM chirp signal is described by the following equation:

(39) $$x\left(t\right)=\sin\left(\phi_{0}+2\pi \left(\frac{ct^{2}}{2}+f_{0}t\right)\right),\;\;t\in \left[0,T\right]$$

with an initial phase of $\phi_{0}$ and represents a signal with frequency varying according to the following linear law:

(40) $$f\left(t\right)=ct+f_{0},\;\;t\in \left[0,T\right],$$

where $f_{0}$ is the initial frequency at time t=0. The rate of rise of *c* is determined by the difference in frequencies $f_{0},f_{1}$ at the initial and final time moments, respectively

(41) $$c=\frac{f_{1}-f_{0}}{T}.$$

The signal simulating the acoustic radiation of a marine vessel is modeled according to [25] as

(42) $$x\left(t\right)=\left(1+\sum_{n=1}^{K}A_{n}\sin\left(2\pi nf_{0}t\right)\right)w_{c}\left(t\right)+w_{e}\left(t\right),$$

where $f_{0}$ is the shaft frequency, i.e., the rotation frequency of the propeller shaft;

$w_{c}\left(t\right),\;w_{e}\left(t\right)$ are the cavitation noise of the propellers and the noise of the marine environment, respectively;

*K* is the number of harmonic components with the fundamental frequency $f_{0}$ forming the signal;

$A_{n}$ are the corresponding amplitudes of each component.

Marine environment noise $w_{e}\left(t\right)$ is modeled with white Gaussian noise with parameters such as to satisfy a predefined signal-to-noise ratio (SNR). The noise of the shaft and propeller rotation is modulated at the shaft rotation frequency $f_{0}$ and at the frequency $mf_{o}$, equal to the product of the shaft rotation frequency and the number of *m* propeller blades (blade frequency) [26]. Due to the nonlinear effects that occur during acoustic radiation, the ship’s noise spectrum, as a rule, contains harmonics of the shaft and blade frequencies, forming a single tonal scale with a base equal to the shaft frequency. In some cases, the shaft frequency and its harmonics may not appear, and then the spectrum may contain only a bladed scale.

Cavitation is the process of formation of discontinuities in the medium during rotation of the propeller, characterized by the appearance of vapor-gas bubbles of various sizes and concentrations in the liquid. Cavitation noise $w_{c}\left(t\right)$ is modeled with white Gaussian noise in a narrow band of cavitation frequencies (from 1 kHz to 3 kHz).

### 6.2. Description of Real Signals

Three datasets corresponding to different signal types will be used as real signals:

- Bioacoustic signals;
- Recordings of hydroacoustic background marine noise;
- Hydroacoustic ship signals.

Whale recordings from the dataset of the article [27] were used as bioacoustic signals. The recordings are three-second segments containing phonemes of whale sounds.

The recordings of natural background noise are taken from the dataset QiandaoEar22 [28], recorded at night in calm conditions on the Chinese Tsandao Lake. Despite the recording conditions, the acoustic signals obtained are non-trivial in terms of spectral content and are very different from, for example, synthetic white noise.

The ship records are sourced from the Deepship [29] dataset, which is the most popular hydroacoustic dataset for solving classification problems using machine learning methods. The dataset was recorded from 2016 to 2018 in Vancouver Bay. The data in this set is divided into four classes: cargo ship, tugboat, tanker, and passenger ship. The advantage of this set is that it is recorded in a marine environment in different seasons and under different conditions. Along with ship signals, the recordings contain natural background noises, sounds of marine mammals, and noises from other human activities. The distance from the objects ranges from several hundred to two thousand meters.

### 6.3. Statistical Experiments for Detecting Model Signals

To illustrate the analytical results of the article, we use a statistical modeling technique based on the analysis of generated numerical data and described in detail in our previous works [22,23]. All numerical results were obtained using Python 3.12 and Numpy 1.26 and Scipy 1.16 libraries. The spectrograms are computed using the AudioFlux [30] digital signal processing library.

Let us consider pairs of data sequences corresponding to two hypotheses of signal reception:

(43) $$\left\{\begin{array}{c} \Gamma_{0}:x_{n}=w_{n},\\ \Gamma_{1}:x_{n}=s_{n}+w_{n},n=1,\ldots ,N. \end{array}\right.$$

The hypothesis $\Gamma_{0}$ corresponds to a decision of receiving only noise, and the hypothesis $\Gamma_{1}$—of receiving a mixture of a useful signal and noise, where the sequence $\{x_{n}\},n=1,\ldots ,N$ is a time series of received data, $\left\{s_{n}\right\}$—useful signal, $\left\{w_{n}\right\}$—additive white Gaussian noise, *N*—the length of the time series of data (i.e., of the frame).

To verify the quality of the separation of the useful deterministic signal and noise, statistics were collected on Q=20,000 numerically generated frames of $\left\{x_{n}\right\}$ signal–noise mixture of length 2*N* = 16,384 with a spectrum size of N=8192, respectively, for each type of signal described in Section 6.1. Spectrograms were computed with windows of size $N_{W}=512$ and hopping length $N_{W}/4$ with Hann window function. Order of Rényi entropy is chosen as α=3.

In all experiments, the $\left\{s_{n}\right\}$ signal for each pair was generated randomly. Thus, for harmonic signals, the number of components K∈[20,50] with random phases varied. For LFM chirp signals, the initial and final frequencies in the window were randomly selected. The additive white Gaussian noise $\left\{w_{n}\right\}$ was obtained by a Gaussian sequence generator with mean μ=0 and variance Σ (within a single set of *Q* frames). The signal amplitude was selected to satisfy a predefined signal-to-noise ratio (SNR), which is described by the formula

(44) $$SNR=10\log_{10}\left(\frac{E_{signal}}{E_{noise}}\right),$$

where $E_{signal}$, $E_{noise}$ are total energies of the signal and noise, respectively, calculated as the sum of the spectral decomposition powers of the sequences $\left\{s_{n}\right\}$ and $\left\{w_{n}\right\}$.

For each resulting sequence $\left\{x_{n}\right\}$, discrete normalized frequency distributions $P_{k}$ and time-frequency distributions $P_{n,k}$ are calculated. Further, based on these distributions, the values of the information characteristics (36) and (37) are calculated for noise and a mixture of noise with a signal corresponding to the two hypotheses from the expressions (43).

The final result of modeling and comparison of the calculated information metrics is the dependence of the quality of the binary classification AUCROC and the detection probability $Pr_{d}$ on the signal-to-noise ratio SNR.

**Figure 1:**
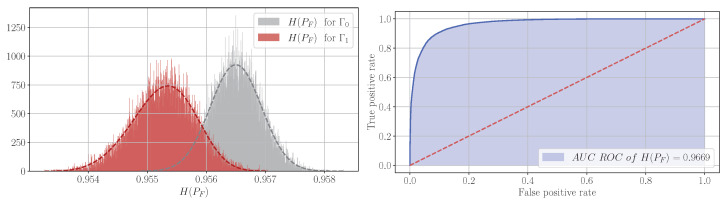
Histogram and AUC ROC graph for $H(P_{F})$, calculated for *Q* = 20,000 experiments.

To obtain such a dependence for a number of Σ noise variance values corresponding to a set of SNRs from −20 dB to 0 dB, the *Q* frame statistics described above are collected, and histograms of information feature distributions are constructed from it, which are then used to calculate the values of AUCROC and the detection probability $Pr_{d}$. An example histogram and AUC ROC graph are shown in Figure 1.

In all the figures presented below, only the most revealing information characteristics are retained in order to simplify the visual understanding of the graphs and get rid of unrepresentative results associated with calculation errors for low SNRs. This approach has no effect on the qualitative conclusions reached at the end of the subsection.

**Figure 2:**
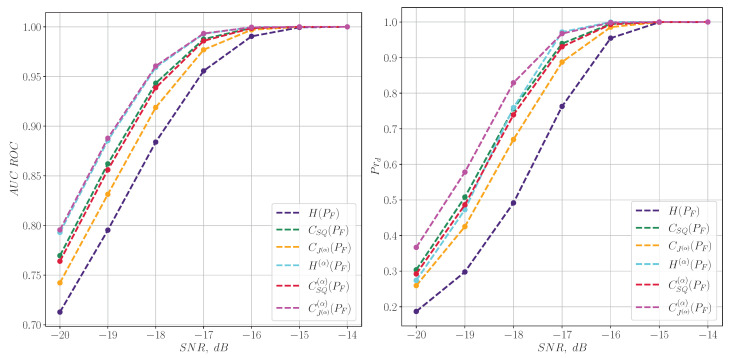
The quality of harmonic signal detection for information characteristics calculated from the spectrum.

Figure 2 shows a comparison of the AUC ROC metric and the probability of detection $Pr_{d}$ for harmonic signals (38) for spectral information characteristics.

**Figure 3:**
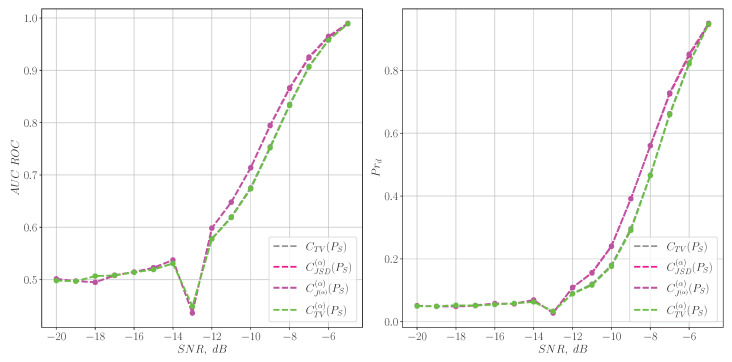
The quality of harmonic signal detection for information characteristics calculated from a spectrogram.

Figure 3 shows a comparison between the AUC ROC metric and the detection probability $Pr_{d}$ for harmonic signals, focusing on information characteristics calculated from a spectrogram. It can be seen that in the case of pure harmonic signals, the spectral characteristics show themselves much better than their time-frequency counterparts.

**Figure 4:**
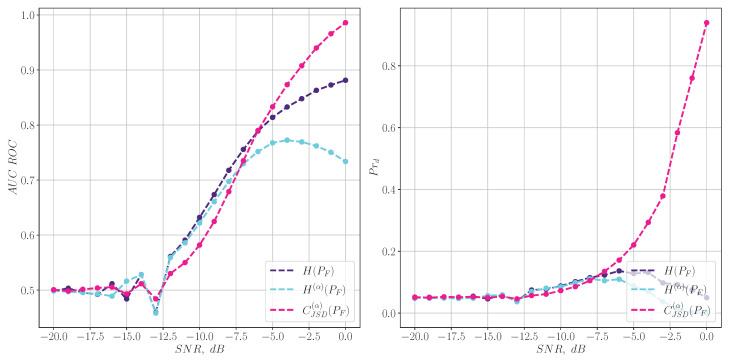
The quality of the LFM chirp signal detection for information characteristics calculated from the spectrum.

**Figure 5:**
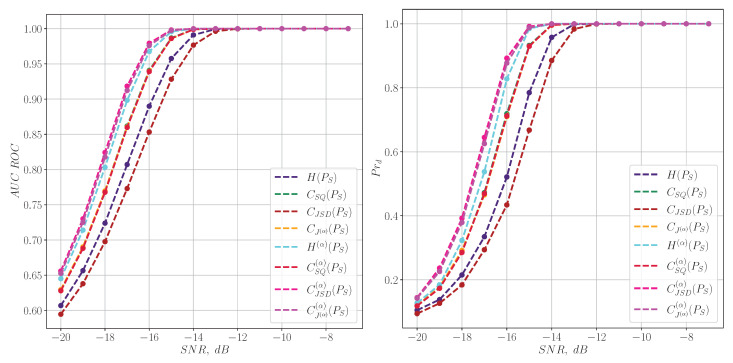
The quality of the LFM chirp signal detection for the information characteristics calculated from the spectrogram.

In turn, the reverse situation is observed for LFM chirp signals (39). In this case, the spectral characteristics degrade significantly even with significant signal-to-noise ratios, as shown in Figure 4, whereas their time-frequency counterparts show good detection quality, as illustrated in Figure 5.

**Figure 6:**
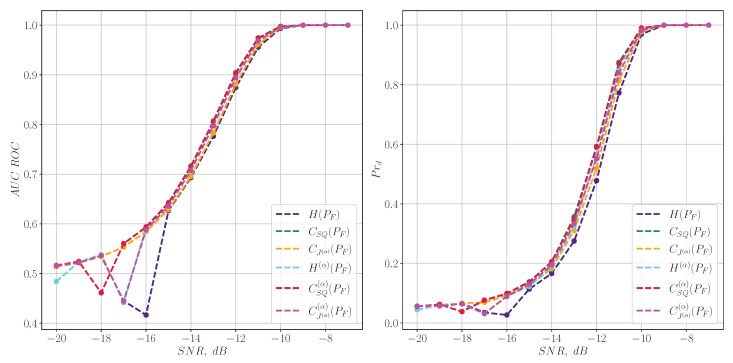
The quality of the simulated acoustic radiation of a marine vessel signal detection for information characteristics calculated from the spectrum.

**Figure 7:**
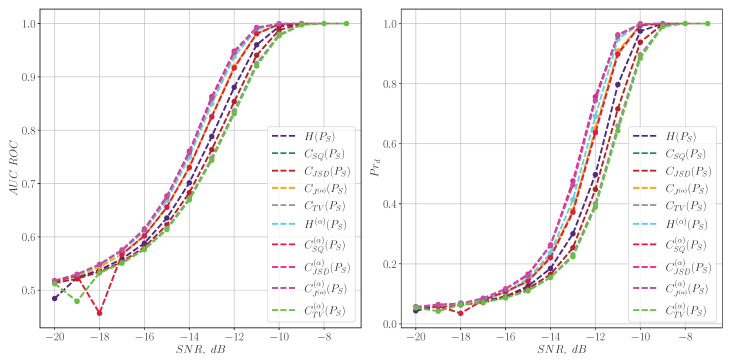
The quality of the simulated acoustic radiation of a marine vessel signal detection for the information characteristics calculated from the spectrogram.

A similar experiment conducted for model cavitation signals of marine vessels (42) demonstrates similar detection quality for both types of information characteristics, as shown in Figure 6 and Figure 7.

The presented graphs correspond to the intuitive idea that time-frequency distributions and features based on them are able to distinguish signals whose frequency composition changes significantly over a time equal to the duration of the window under consideration.

Therefore, in the case of simple harmonic signals, the constant components have a positive effect on the spectral characteristics, since the spectral window is trivially larger than the atomic windows used to calculate the spectrogram, and thus shows adequate signal–noise separation quality for lower SNRs.

However, in the case of LFM chirp signals, their spectral composition changes during the observation window, i.e., the frequency transformation from the entire window is no longer able to adequately reflect the signal features, whereas in the time-frequency distribution matrix, frequencies are localized much better and therefore features based on time-frequency transformations stand out favorably in the problem of detecting such signals.

**Figure 8:**
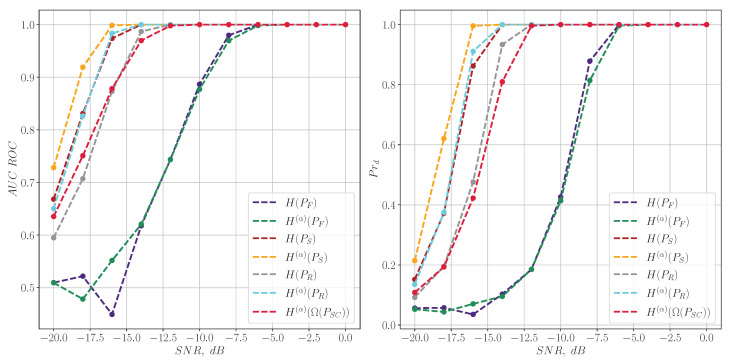
The quality of LFM chirp signal detection for different entropy measures.

Lastly, entropy characteristics based on $P_{SC}$ distribution (19) are compared with other criteria. This comparison for LFM chirp signals is illustrated in Figure 8. It can be seen that $H^{\left(\alpha \right)}\left(\Omega \left(P_{SC}\right)\right)$ (20) distribution’s detection quality is between the ones related to spectral and spectrogram-based characteristics, which can be of research interest and may be studied in future work.

### 6.4. H/C Plane for Classification of Real Signals

Next, we will consider the real signals from the datasets described in Section 6.2. It is worth noting that the recordings were not subjected to any pre-processing, except for resampling to a unite frequency, which is necessary for the uniformity of the size of the signal windows of all classes of the training dataset, as well as centering and normalization, which are standard procedures in the practice of working with acoustic signals using machine learning methods.

Entropy characteristics are associated with an effective signal classification mechanism based on the analysis of the H/C plane [31,32]. Without going into the details outlined in the mentioned articles, this approach is justified by the fact that the complexity estimate provides additional insight into the details of the probability distribution of the system, which are not distinguished by entropy. It can also help reveal information related to the correlation structure between the components of the studied physical process [31]. Hence, the entropy–complexity plane H/C allows you to explore the hidden parameters of the signals [6] and can be used to classify them.

**Figure 9:**
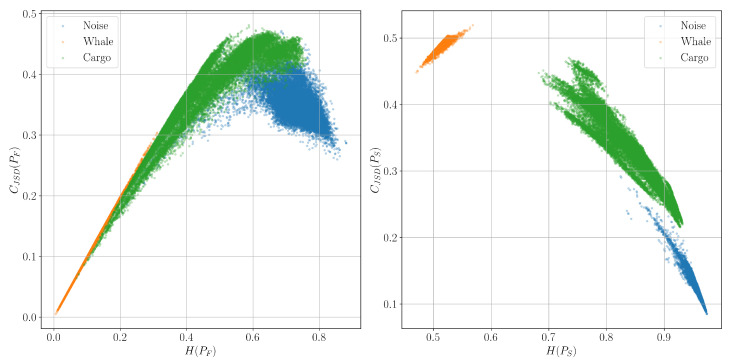
Classification planes for the Shannon entropy *H* and the corresponding complexity $C_{JSD}$ for the spectrum $P_{F}$ and spectrogram $P_{S}$ distributions.

**Figure 10:**
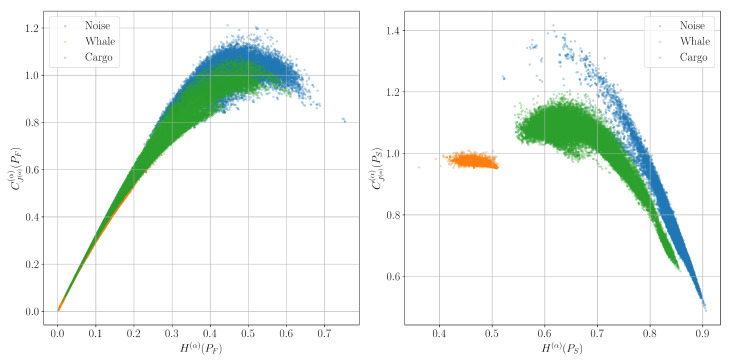
Classification planes for the Rényi entropy $H^{\left(\alpha \right)}$ and the corresponding complexity $C_{J^{\left(\alpha \right)}}^{\left(\alpha \right)}$ for the spectrum $P_{F}$ and spectrogram $P_{S}$ distributions.

**Figure 11:**
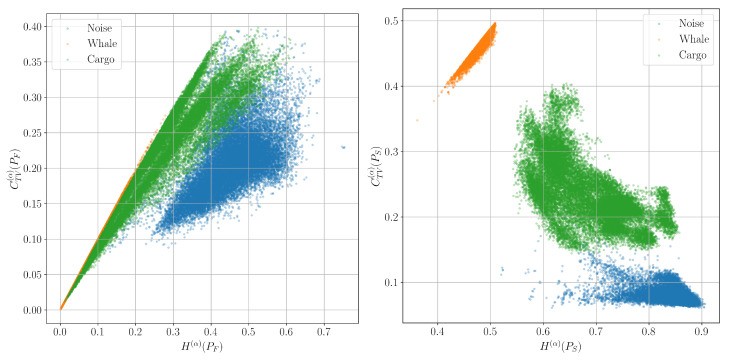
Classification planes for the Rényi entropy $H^{\left(\alpha \right)}$ and the corresponding complexity $C_{TV}^{\left(\alpha \right)}$ for the spectrum $P_{F}$ and spectrogram $P_{S}$ distributions.

Figure 9, Figure 10 and Figure 11 show such planes constructed from signals corresponding to the classes of background marine noise, cargo ship, and biacoustic signatures of whales. The approximate number of signal windows of each class, and, accordingly, the colored dots of each color on the graphs in this experiment is 20,000.

It can be seen that while the spectral characteristics do not allow us to reliably separate the described signal classes, the time-frequency characteristics successfully cope with this task.

**Figure 12:**
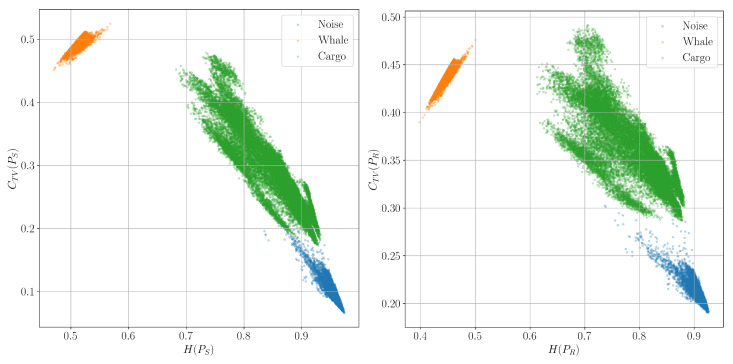
Classification planes for the Shannon entropy *H* and the corresponding complexity $C_{TV}$ for conventional $P_{S}$ and reassigned spectrograms $P_{R}$ distributions.

In addition, it is worth noting how different time-frequency distributions affect the appearance of such diagrams. Figure 12 shows the H/C planes constructed for the usual (7) and reassigned (11) spectrograms. It can be seen that clouds of points corresponding to different classes are more strongly grouped around their centers of mass, and the distances between the different classes become greater.

The demonstrated results show that information features of frequency and time-frequency distributions of signals can be used to solve the problem of signal classification, which is the subject of the next subsection.

### 6.5. Using Entropy Features to Classify Signals with Machine Learning Methods

To study the possibilities of classifying real signals using the proposed features and numerically evaluating the quality of such classification, a machine learning method was used, namely the XGBoost [33] gradient boosting algorithm for decision trees, which is one of the most popular approaches for solving machine learning problems based on tabular data. The results were obtained using the XGBoost library of the Python programming language.

For each class of signals, a training dataset has been calculated, which has the size of 20,000 signal windows for 30 features for each class. It is important that the training and test datasets are separated before the stage of feature calculation, i.e., it is guaranteed that the test dataset contains signals that are not present in the training dataset. The test dataset contains 5000 signal windows for each class. Four classes are considered in the experiment:

- natural marine background noise (Noise);
- bioacoustic signals of whales (Whale);
- hydroacoustic signals of a tugboat (Tug);
- hydroacoustic signals of a passenger ship (Passenger).

**Figure 13:**
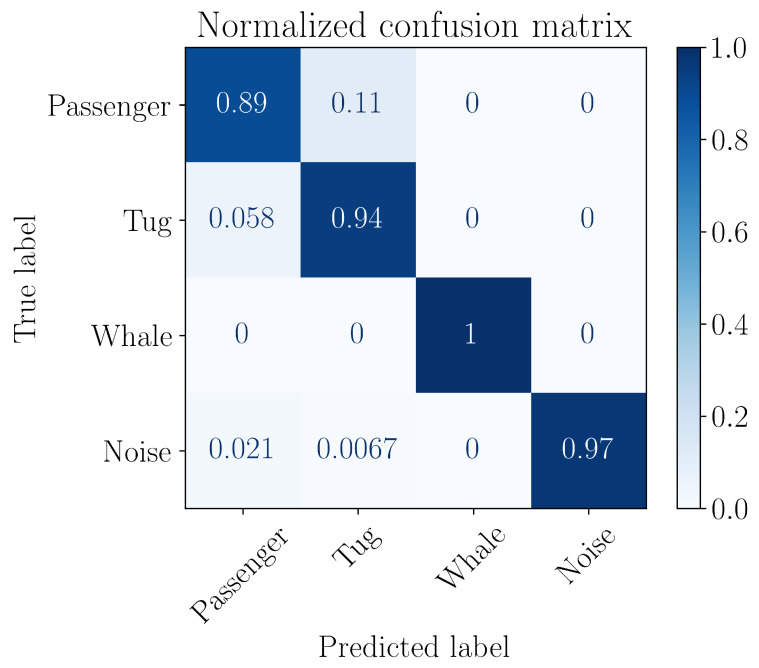
Confusion matrix of the trained classifier.

As a result of training the classifier, the following metrics were obtained:

(45) $$\begin{array}{c} Accuracy=0.95,\\ Macro\;F1=0.95,\\ MCC=0.93, \end{array}$$

where MacroF1 is a F1 measure averaged over all classes, and MCC is the Matthews correlation coefficient. Figure 13 shows a normalized error matrix after training the model.

**Figure 14:**
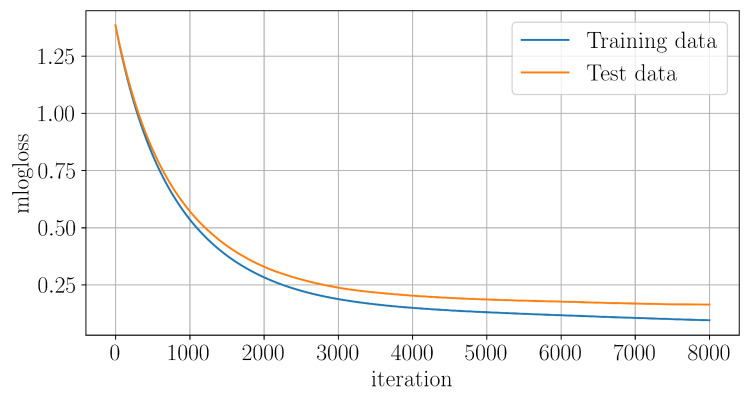
XGBoost classifier training graph.

Figure 14 shows a training performance graph, i.e., the dependence of the loss function mlogloss (multiclass logarithmic loss) for training and test data from iteration. A high final quantity of the obtained trees is associated with the chosen learning step lr=0.001, which guarantees the smoothness of the gradient convergence process.

**Figure 15:**
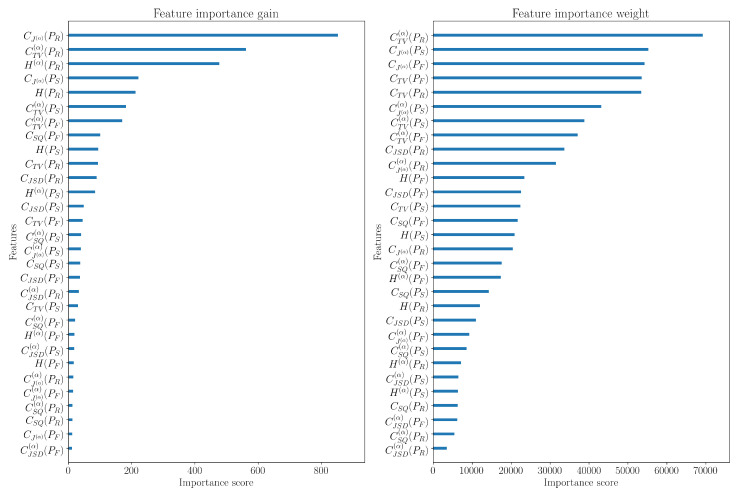
The importance of features in classification.

Furthermore, it is interesting to examine the significance of the trained classifier’s features. From Figure 15, the influence of the reassigned spectrogram distribution $P_{R}$ for making the final decision becomes obvious, since the features associated with it hold the highest importance and weight in decision-making by the received classifier.

Thus, it can be stated that information features, considered in the current work, achieve good classification quality for acoustic signals of different natures and can be used in machine learning detection and classification systems.

## 7. Conclusions

The paper provides a time-frequency analysis of the problem of detecting and classifying acoustic signals based on information (entropy) criteria. A new method for calculating the discrete distribution in the time-frequency domain is proposed, including the use of a spectrogram and a reassigned spectrogram. Further information properties of the Ω matrix and the Π vector in the problem of distinguishing close hypotheses for weak signal detection have yet to be established.

To justify the applicability of the proposed characteristics and validate their classification quality, modeling on synthetic signals and numerical verification of the solution of the multiclass classification problem based on machine learning methods on real hydroacoustic recordings are carried out. The obtained high classification results (F1=0.95) confirm the potential of using the proposed characteristics.

Future work will be devoted to an additional study of the problem of classifying similar classes of signals, as well as the joint use of the proposed information characteristics and classical signal features to improve the quality of classification.

## Data Availability

The original data presented in the study are openly available in https://www.frdr-dfdr.ca/repo/dataset/4a3113e6-1d58-6bb4-aaf2-a9adf75165be (accessed on 10 August 2025), https://github.com/irfankamboh/DeepShip (accessed on 10 August 2025) and https://github.com/xiaoyangdu22/QiandaoEar22 (accessed on 10 August 2025).

## Abbreviations

The following abbreviations are used in this manuscript:

AUC ROC	Area Under the Receiver Operating Characteristic Curve
CFAR	Constant False Alarm Rate
ECG	Electrocardiogram
EEG	Electroencephalogram
JSD	Jensen–Shannon Divergence
LFM	Linear Frequency Modulation
SNR	Signal-To-Noise Ratio
STFT	Short-Time Fourier transform
TFD	Time-Frequency Distribution
VAD	Voice Activity Detection
